# 2-arachidonyl glycerol modulates astrocytic glutamine synthetase via p38 and ERK1/2 pathways

**DOI:** 10.1186/s12974-018-1254-x

**Published:** 2018-08-03

**Authors:** Shenghong Wang, Hua Zhang, Bin Geng, Qiqi Xie, Wenzhou Li, Yajun Deng, Weidong Shi, Yunyan Pan, Xuewen Kang, Jing Wang

**Affiliations:** 1 Key Laboratory of Orthopaedics Disease of Gansu Province, Lanzhou University Second Hospital, No.82 Cuiyingmen Street, Lanzhou, Gansu 730030 People’s Republic of China; 2 Department of Orthopaedics, Lanzhou University Second Hospital, No.82 Cuiyingmen Street, Lanzhou, Gansu 730030 People’s Republic of China; 3 Clinical Laboratory, Lanzhou University Second Hospital, No.82 Cuiyingmen Street, Lanzhou, Gansu 730030 People’s Republic of China

**Keywords:** 2-AG, Astrocyte, Glutamine synthetase, MAPK, p38, ERK

## Abstract

**Background:**

The glutamine synthetase (GS), an astrocyte-specific enzyme, is involved in lipopolysaccharide (LPS)-induced inflammation which activates the mitogen-activated protein kinase (MAPK) signaling. Endocannabinoid 2-arachidonyl glycerol (2-AG) has been described to serve as an endogenous mediator of analgesia and neuroprotection. However, whether 2-AG can directly influence astrocytic GS and MAPK expressions remains unknown.

**Methods:**

In the present study, the effects of 2-AG on astrocytic GS expression, p38 and ERK1/2 expression, cell viability, and apoptosis following LPS exposure were investigated.

**Results:**

The results revealed that LPS exposure increased GS expression with p38 activation in the early phase and decreased GS expression with activation of ERK1/2, decrease of cell viability, and increase of apoptosis in the late phase. Inhibition of p38 reversed GS increase in the early phase while inhibition of ERK1/2 reversed GS decrease in the late phase induced by LPS exposure. 2-AG protected astrocytes from increase of apoptosis and decrease of cell viability induced by the late phase of LPS exposure. In the early phase of LPS exposure, 2-AG could suppress the increase of GS expression and activation of p38 signaling. In the late phase of LPS exposure, 2-AG could reverse the decrease of GS expression and activation of ERK1/2 induced by LPS.

**Conclusion:**

These findings suggest that 2-AG could maintain the GS expression in astrocytes to a relatively stable level through modulating MAPK signaling and protect astrocytes from LPS exposure.

## Background

Astrocytes are the main glial cells in the central nerve system (CNS) where they play a central role in neurotrophic support, regulation of the concentration of extracellular ions, antioxidant defense, and neurotransmitter metabolism [1]. In astrocytes, glutamine synthetase (GS), an ATP-dependent enzyme, could modulate the extracellular level of glutamate, an essential but neurotoxic excitatory neurotransmitter, by converting glutamate to nontoxic amino acid glutamine [2]. Changes in GS expression have been identified in a number of neurological disorders, including traumatic brain injury, neurodegenerative diseases, and various models of nociceptive pain, while modulating GS expression could diminish relative clinical features [3–8]. Endocannabinoids are endogenous lipid signaling mediators capable of modulating synaptic function and producing neuroprotection and anti-inflammation [9, 10]. 2-arachidonoyl glycerol (2-AG), the most abundant endocannabinoid, has been illustrated to play a significant role in protecting neurocytes from inflammatory injuries, such as stimuli of interleukine-1 beta (IL-1β), lipopolysaccharide (LPS), and β-amyloid in models of neurodegenerative diseases [11, 12]. Besides, 2-AG has been shown to have the ability of controlling neuropathic pain and mechanical hyperalgesia in several preclinical models of chronic pain [13–16]. Although the role of 2-AG has been extensively investigated, it is not clear whether 2-AG could directly modulate the GS expression in astrocytes, and the exact molecular mechanism remains unknown.

The mitogen-activated protein kinase (MAPK) cascades are a family of serine/threonine kinases that can mediate a wide variety of extracellular stimuli into the cytoplasm and nuclei and regulate cellular gene expression and protein synthesis [17]. Previous studies have indicated that MAPK members, extracellular signal-regulated protein kinase 1/2 (ERK1/2) and p38 pathways, in astrocytes may be involved in the process of various neurological disorders [18]. Accumulating evidence showed that activation (phosphorylation) of ERK1/2 and p38 in spinal astrocytes under different persistent pain conditions results in the generation and maintenance of pain hypersensitivity via distinct molecular and cellular mechanisms [19]. In addition, activation of ERK1/2 and p38 in cortical astrocytes has been identified in several neurodegenerative diseases, while blockade of ERK1/2 and p38 pathways has been shown to alleviate inflammation and clinical features in different animal models [20–22].

It has been verified that 2-AG could modulate synaptic function, produce neuroprotection, and stimulate MAPK family by bonding to and activating two receptors, CB_1_R and CB_2_R, which are two G-protein-coupled receptors and have been grossly identified in astrocytes [23]. Previous studies have shown that activation of CB_1_R or CB_2_R results in anti-inflammation, prevention of neurodegeneration, and inhibition of nociceptive signaling pathways [24–26], as well as stimulation of the MAPK cascade [27–29]. Interestingly, activation of CB_1_R or CB_2_R also could inhibit stress-induced activation of the MAPK cascade. Based on these findings, it is proposed that 2-AG may exert neuroprotection and analgesia via activating CB_1_R or CB_2_R and regulating activation of the MAPK cascade.

## Methods

### Primary astrocyte cultures

Primary astrocytes from the cerebral cortex of neonatal Sprague–Dawley rats (postnatal 1~3 days) were cultured as described preciously [30]. The neonatal Sprague–Dawley rats were provided by the Experimental Animal Center of Gansu University of Chinese Medicine, China. All efforts were performed to minimize the number of neonatal rats used and their suffering. The procedures were approved by the Animal Care and the Ethic Committee of Animal Usage of Lanzhou University Second Hospital. Briefly, the newborn rats were decapitated and the cerebral hemispheres were aseptically removed into HBSS (Hank’s Balanced Salt Solution). After removal of the meninges, the cerebral cortices were cut into small pieces, digested with 0.25% Trypsin-EDTA (Gibco Life Technology, CA, USA), mechanically dissociated by gentle pipetting with Pasteur pipette, and then centrifuged at 400*g* for 5 min. The cells were resuspended in complete culture medium containing 90% DMEM/F12 (Gibco Life Technology, CA, USA) and 10% FBS (PAN-Biotech, Germany), and plated at a density of 3~5 × 10^5^ cells/cm^2^ in 25-cm^2^ flasks. Cells in flasks were cultured at 37 °C in CO_2_ incubator for 5~7 days to reach the first confluence. To obtain quite pure astrocytes (more than 95%), the confluent cultures in flasks were shaken at 200 rpm overnight to diminish microglia contamination. Afterward, the astrocytes were evenly passaged into 35-mm dishes for western blot analysis, on coverslips pre-coated with poly-l-lysine for immunocytochemistry analysis, and into 96-well plates for methyl thiazolyl tetrazolium (MTT) analysis after different treatments. Astrocytes were cultured with serum-free medium for 6 h before different treatments.

### Drugs treatment

All drugs were dissolved and/or diluted with serum-free DMEM/F12 into final concentration. To study the effect of 2-AG, astrocytes were incubated with 0.01 μM 2-AG for 2 h and then stimulated with 1 μg/ml LPS. To study the roles of ERK1/2 and p38 in LPS-induced inflammation, astrocytes were pretreated with PD98059 or SB203580 for 1 h before LPS exposure. To study the roles of CB_1_R and CB_2_R on effects of 2-AG, astrocytes were pretreated with 1 μM AM281 or AM630 for 1 h before the treatment of 2-AG and LPS.

### Hoechst 33342 staining

After various treatments, astrocytes were stained with Hoechst 33342 kit (St. Louis, MO, USA) and counted blindly as described previously [31]. Briefly, astrocytes on coverslips were rinsed with PBS and fixed with 4% paraformaldehyde for 30 min. After being rinsed three times with PBS, astrocytes were incubated with 0.4% Triton X-100 for 20 min, and then stained with Hoechst 33342 for 10 min in the dark. After washing cells with PBS, the nuclear morphological changes of apoptosis were observed using a fluorescence microscope (Olympus, Japan). Astrocytes with bright staining, highly condensed and fragmented nuclei were defined as apoptotic cells. The number of apoptotic cells and total cells were counted, and then the cell apoptosis rate was calculated by the following equation:

$$ \mathrm{Cell}\ \mathrm{apoptosis}\ \mathrm{rate}\ \left(\%\right)=\left({N}_{\mathrm{apoptotic}\ \mathrm{cells}}/{N}_{\mathrm{total}\ \mathrm{cells}}\right)\times 100 $$

### Methyl thiazolyl tetrazolium (MTT) assay

Cell viability was detected using 3-(4,5-dimethythiazol-2-yl)-2,5-diphenyl-tetrazolium (MTT) assay as described previously [31]. In brief, astrocytes were cultured in 96-well plates at a density of 3 × 10^4^ cells/well. After different treatments, astrocytes were incubated with 1 mg/ml substrate MTT at 37 °C for 4 h. Then the culture medium was replaced by 100 μl dimethyl sulfoxide (DMSO) to dissolve the formazan crystals. The amount of formazan was measured at 570 nm using a Universal Microplate Reader (Elx 800; Bio-TEK instruments, Winooski, VT, USA). The cell viability was expressed as a percentage of viable cells in treated groups versus a control group using the following formula:

$$ \mathrm{Cell}\ \mathrm{viability}\ \left(\%\right)=\left({\mathrm{Opticaldensity}}_{\mathrm{treatment}}/{\mathrm{Opticaldensity}}_{\mathrm{control}}\right)\times 100 $$

### Protein isolation and western blotting

Western blotting was performed according to a previous report [32]. Briefly, astrocytes in 35-mm dishes were lysed with 100 μl radioimmunoprecipitation assay (RIPA) lysis buffer containing 1% phenylmethanesulfonyl fluoride (PMSF) after different treatments. The lysates were centrifuged at 12,000 rpm for 10 min to clear cell debris and further diluted with 30 μl sample buffer. Total protein in lysates was loaded onto 10% SDS-polyacrylamide gels at 5–20 μg per lane (as measured by BCA), then separated by electrophoresis and transferred to PVDF membranes. Following the blockade of nonspecific binding sites with 5% non-fat milk in Tris-buffered saline with Tween-20 (TBST) for 2 h at room temperature (RT), the membranes were incubated overnight at 4 °C with primary antibodies according to the manufacturer’s instruction (anti-MAPK antibody, 1:1000, Cell Signaling Technology, MA, USA; anti-GS antibody, 1:10,000, St. Louis, MO, USA) and then washed extensively with TBST three times at 10-min intervals and incubated with appropriate second antibodies (1:10,000; Danvers, MA, USA) at RT for 2 h. The membranes were washed three times with TBST at 10-min intervals, and immunolabeled protein bands on membranes were detected using an enhanced chemiluminescence kit.

### Immunocytochemistry

After different treatments, the astrocytes on coverslips were fixed with 4% paraformaldehyde for 30 min and washed with PBS. The fixed cells were permeabilized with 0.4% TritonX-100 for 20 min, washed again with PBS, blocked with 3% normal goat serum for 30 min, and then incubated with different primary antibodies (GS, 1:5000; ERK1/2, 1:500; p38, 1:500) overnight at 4 °C, respectively. After 24 h, the coverslips were washed and incubated with appropriate second antibodies (Invitrogen, UK) conjugated with Alexa Fluor® 488 (green staining) or 594 (red staining) for 2 h at RT. All cells were stained with DAPI for nuclei observation, and cells were visualized by an immunofluorescence microscope (Olympus, Japan).

### Statistics analysis

All experiments were carried out in triplicate and repeated at least three times, and all measurements were performed by blinded evaluators. The data were expressed as mean ± standard deviation (SD), and STATA software (version 14.2, Stata Corp, College Station, TX, USA) was used for statistical analysis. One-way ANOVA followed by Neuman Keuls test was mainly performed, and two-way ANOVA followed by Dennett test was performed in Fig. 4; *p* < 0.05 was set as the level of significant difference.

## Results

### LPS activated rat primary astrocyte dosage-dependently and GS time-dependently

**Fig. 1:**
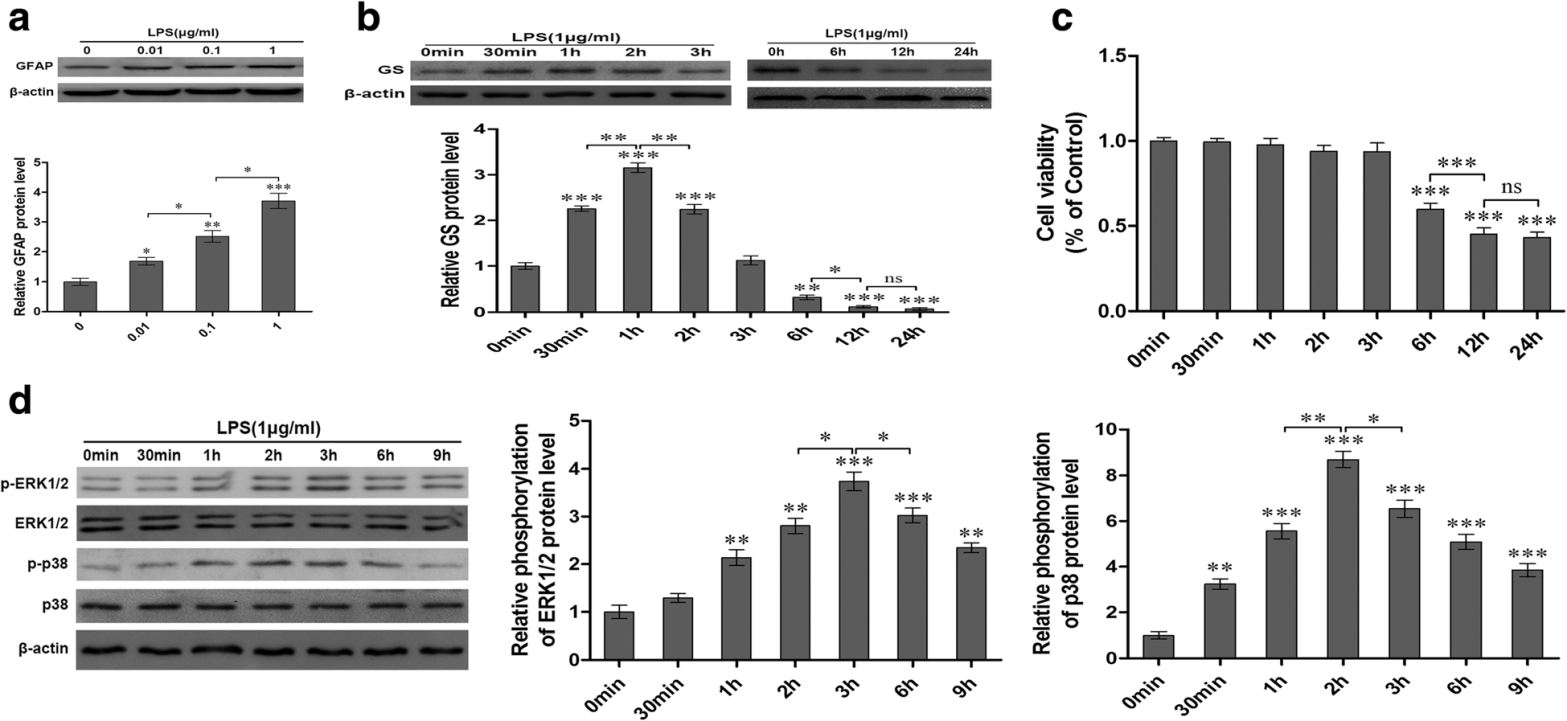
LPS exposure induced activation of astrocytes (**a**), changes of GS expression in astrocytes (**b**), decrease of cell viability in astrocytes (**c**), and activation of MAPK (**d**). Astrocytes were treated with LPS at different dosages for 6 h and GFAP expression was analyzed (**a**). Then astrocytes were treated with 1 μg/ml LPS for different times and GS expression (**b**), cell ability (**c**), and activation of MAPK were analyzed (**d**). Error bars were ± SD. *n* = 3. *p < 0.05, **p < 0.01 and ***p < 0.001 versus control

Astrocyte activation is a common outcome of neurological disorders. In the present study, LPS was used to activate astrocytes. Cells were incubated in DMEM/F12 containing LPS at various concentrations (0, 0.01, 0.1, and 1 μg/ml) for 6 h, and GFAP expression was analyzed using western blotting to assess the activation of astrocytes (Fig. 1a). The data showed that compared with control (1.00 ± 0.12 for 0 μg/ml of LPS), the expression of GFAP in astrocytes with LPS treatment was significantly increased in a dose-dependent manner (1.68 ± 0.13 for 0.01 μg/ml, *p* < 0.05; 2.52 ± 0.20 for 0.1 μg/ml, *p* < 0.01; 3.71 ± 0.25 for 1 μg/ml, *p* < 0.001).

In order to investigate the effects of LPS exposure on GS expression, astrocytes were exposed to 1 μg/ml LPS for different times and the level of GS expression was assessed using western blotting (Fig. 1b). The results indicated that LPS exposure induced time-dependent biphasic changes of GS expression in astrocytes; i.e., compared with control (1.00 ± 0.07 for 0 min), GS expression began to increase at 30 min (2.26 ± 0.06, *p* < 0.001), peaked at 1 h (3.15 ± 0.10, *p* < 0.001), declined to control level at 3 h (1.12 ± 0.10, *p* > 0.05), and then decreased at 6 h (0.32 ± 0.05, *p* < 0.01), 12 h (0.18 ± 0.02, *p* < 0.001), and 24 h (0.06 ± 0.03, *p* < 0.001).

### Late phase of LPS exposure decreased astrocyte viability time-dependently

To investigate the effect of LPS on astrocyte viability, astrocytes were exposed with 1 μg/ml LPS for different times and astrocyte viability was assessed with MTT assay (Fig. 1c). The data indicated that LPS exposure decreased the astrocyte viability at the late phase, i.e., astrocyte viability decreased at 6 h (0.60 ± 0.01, *p* < 0.001), 12 h (0.45 ± 0.01, *p* < 0.001), and 24 h (0.43 ± 0.01, *p* < 0.001) when compared with control (1.00 ± 0.01 for 0 min), while the cell viability of astrocytes at the early phase (30 min, 1 h, 2 h, and 3 h) was not significantly changed (*p* > 0.05, Fig. 1c).

### LPS activated the phosphorylation of p38 and ERK1/2 in astrocytes

To investigate the effects of LPS on MAPK cascade in astrocytes, the expression of p-ERK1/2, ERK1/2, p-p38, and p38 in primary astrocytes was investigated using western blotting after treatment with 1 μg/ml LPS for 0 min, 30 min, 1 h, 2 h, 3 h, 6 h, and 9 h (Fig. 1d). The data showed that LPS exposure significantly increased the phosphorylation of ERK1/2 and p38 in different patterns without effect on the total ERK1/2 and p38. The expression of p-ERK1/2 began to increase at 1 h (2.14 ± 0.17, *p* < 0.01) and gradually reached peak at 3 h (3.74 ± 0.20, *p* < 0.001), then declined but was still higher at 9 h (2.34 ± 0.10, *p* < 0.01) than control. The expression level of p-p38 increased at 30 min (3.24 ± 0.23, *p* < 0.01) and reached to peak at 2 h (8.70 ± 0.35, *p* < 0.001), and then gradually declined but was still higher at 9 h (3.85 ± 0.27, *p* < 0.001) than control.

### 2-AG protected astrocytes from apoptosis and decrease of cell viability induced by the late phase of LPS exposure

**Fig. 2:**
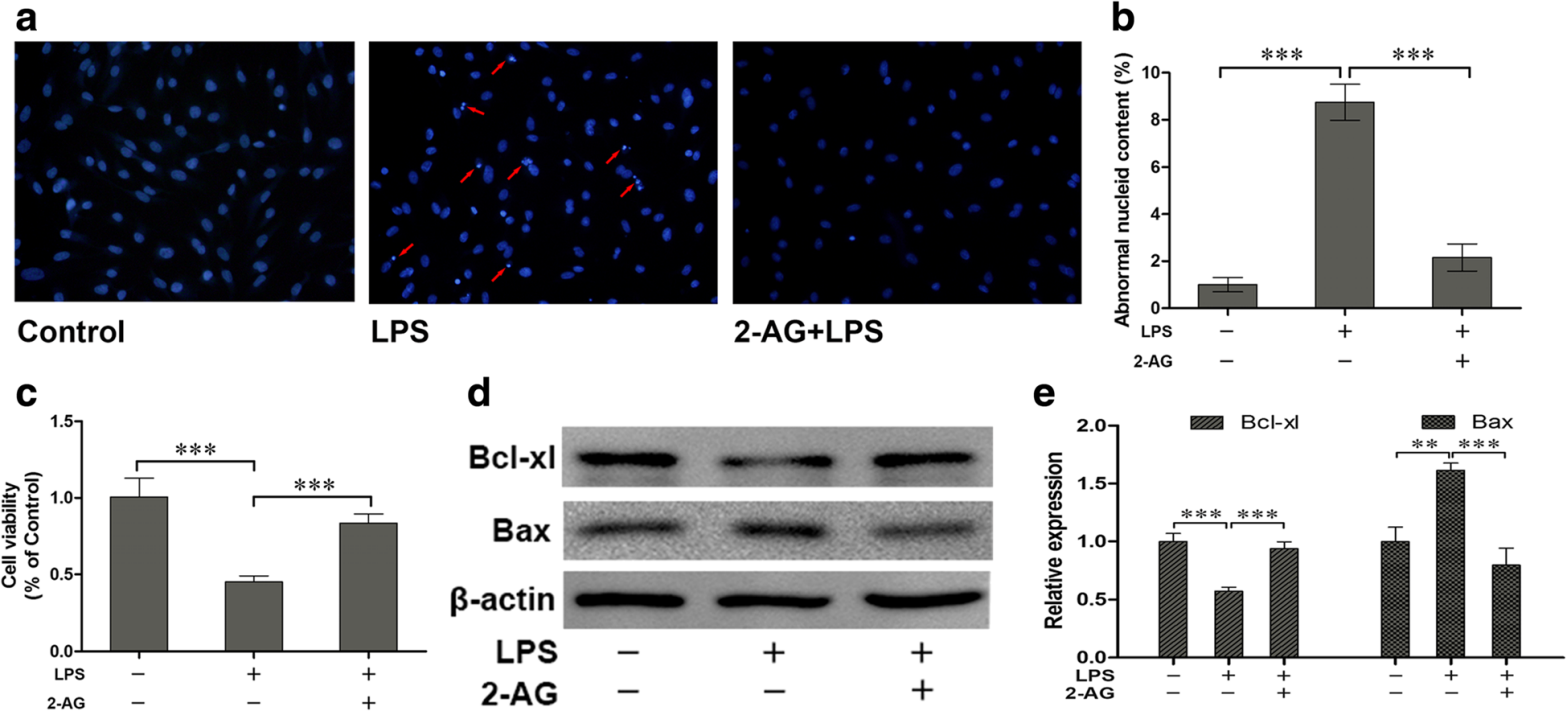
Effects of 2-AG on the change of cell apoptosis and viability of astrocytes induced by LPS. Astrocytes were pretreated with 0.01 μM 2-AG for 2 h and exposed to 1 μg/ml LPS for 12 h. Cell apoptosis was measured by Hoechst 33342 assay (**a**, **b**) (the red arrows indicate the apoptotic cells) and MTT was used to measure cell viability (**c**). The expressions of Bcl-xl and Bax were evaluated through western blotting assay (**d**, **e**). Scar bar = 20 μm, error bars were ± SD. *n* = 3. *p < 0.05, **p < 0.01, and ***p < 0.001

To evaluate the potential protective roles of 2-AG in cultured astrocytes, the astrocytes were pretreated with 0.01 μM 2-AG for 2 h and then exposed to 1 μg/ml LPS for 12 h. The Hoechst 33343 staining (Fig. 2a) and corresponding statistical chart (Fig. 2b) showed that 2-AG significantly attenuated the cell apoptosis induced by LPS exposure (2.16 ± 0.58 for 2-AG plus LPS vs. 8.75 ± 0.77 for LPS alone, *p* < 0.001). Similarly, MTT assay (Fig. 2c) indicated that 2-AG significantly reversed the decrease of cell viability induced by LPS exposure (0.84 ± 0.02 for 2-AG plus LPS vs. 0.45 ± 0.02 for LPS alone, *p <* 0.001). Bax and Bcl-xl are two members of Bcl-2 family of proteins. Bax acts as a pro-apoptotic regulator and Bcl-xl acts as an anti-apoptotic regulator. The western blotting results (Fig. 2d) and corresponding statistical chart (Fig. 2e) showed that 2-AG significantly reversed the decrease of Bcl-xl (0.94 ± 0.04 for 2-AG plus LPS vs. 0.57 ± 0.02 for LPS alone, *p <* 0.001) and the increase of Bax (0.79 ± 0.09 for 2-AG plus LPS vs. 1.61 ± 0.04 for LPS alone, *p <* 0.001) induced by LPS exposure.

### Inhibition of p38 attenuated GS upregulation in astrocytes with the early phase of LPS exposure

**Fig. 3:**
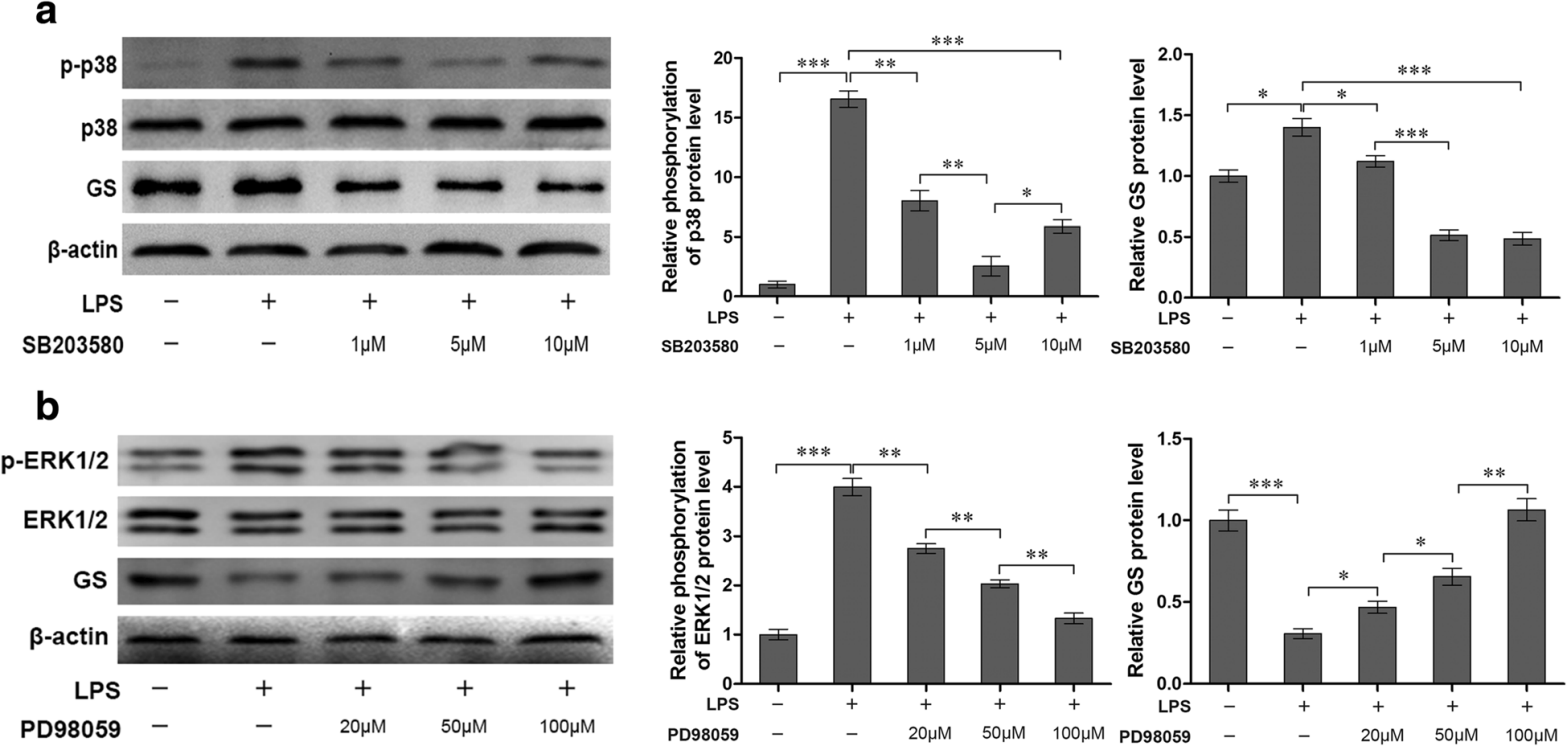
Effects of inhibition of MAPK on the phosphorylation of p38 (**a**) and ERK1/2 (**b**), and GS expression (**a**, **b**). Astrocytes were pretreated with SB203580 (**a**) or PD98059 (**b**) for 1 h and exposed to 1 μg/ml LPS for 2 h (**a**) or 12 h (**b**). The protein level of GS, p-p38 and p-ERK1/2 were measured, respectively (**a**, **b**). Error bars were ± SD. *n* = 3. *p < 0.05, **p < 0.01, and ***p < 0.001

To validate the potential involvement of p38 signaling pathway in the regulation of GS expression in astrocytes, the astrocytes exposed to 1 μg/ml LPS for 2 h was chosen on the basis on the acquired data from Fig. 1. Pretreatment with p38 inhibitor SB203580 at the concentration of 1, 5, and 10 μM for 1 h significantly reversed the phosphorylation level of p38 induced by LPS (8.03 ± 0.85, *p* < 0.01; 2.55 ± 0.82, *p <* 0.001; 5.88 ± 0.57, *p <* 0.001, respectively) when compared with LPS alone group (16.58 ± 0.69, Fig. 3a), and the inhibition of 10 μM LPS was weaker than 5 μM LPS (*p <* 0.01). As expected, SB203580 at the concentration of 1, 5, and 10 μM also suppressed the upregulation of GS induced by LPS exposure at the early phase (2 h) (1.12 ± 0.05, *p <* 0.05; 0.51 ± 0.04, *p* < 0.001; 0.48 ± 0.05, *p <* 0.001, respectively) when compared with LPS alone group (1.40 ± 0.07, Fig. 3a).

### Inhibition of ERK1/2 reversed GS downregulation in astrocytes by the late phase of LPS exposure

To further investigate the effects of ERK1/2 on GS downregulation induced by LPS, LPS exposure for 12 h was chosen in view of data from Fig. 1 and astrocytes were pretreated with ERK1/2 inhibitor PD98059 at the concentration of 20, 50, and 100 μM for 1 h. The results indicated that PD98059 significantly inhibited the phosphorylation of ERK1/2 induced by LPS in a dose-dependent manner (2.74 ± 0.10 for 20 μM, *p* < 0.01; 2.03 ± 0.08 for 50 μM, *p* < 0.01; 1.32 ± 0.11 for 100 μM, *p* < 0.01, respectively) when compared with LPS alone group (3.99 ± 0.18, Fig. 3b). Furthermore, PD98059 pretreatment could reverse the downregulation of GS expression induced by LPS exposure at late phage (0.47 ± 0.04 for 20 μM, *p* < 0.05; 0.66 ± 0.05 for 50 μM, *p* < 0.05; 1.06 ± 0.07 for 100 μM, *p* < 0.01; respectively), in a dose-dependent manner, when compared to LPS alone group (0.31 ± 0.03, Fig. 3b).

### 2-AG suppressed p38 activation and GS upregulation induced by the early phase of LPS exposure

**Fig. 4:**
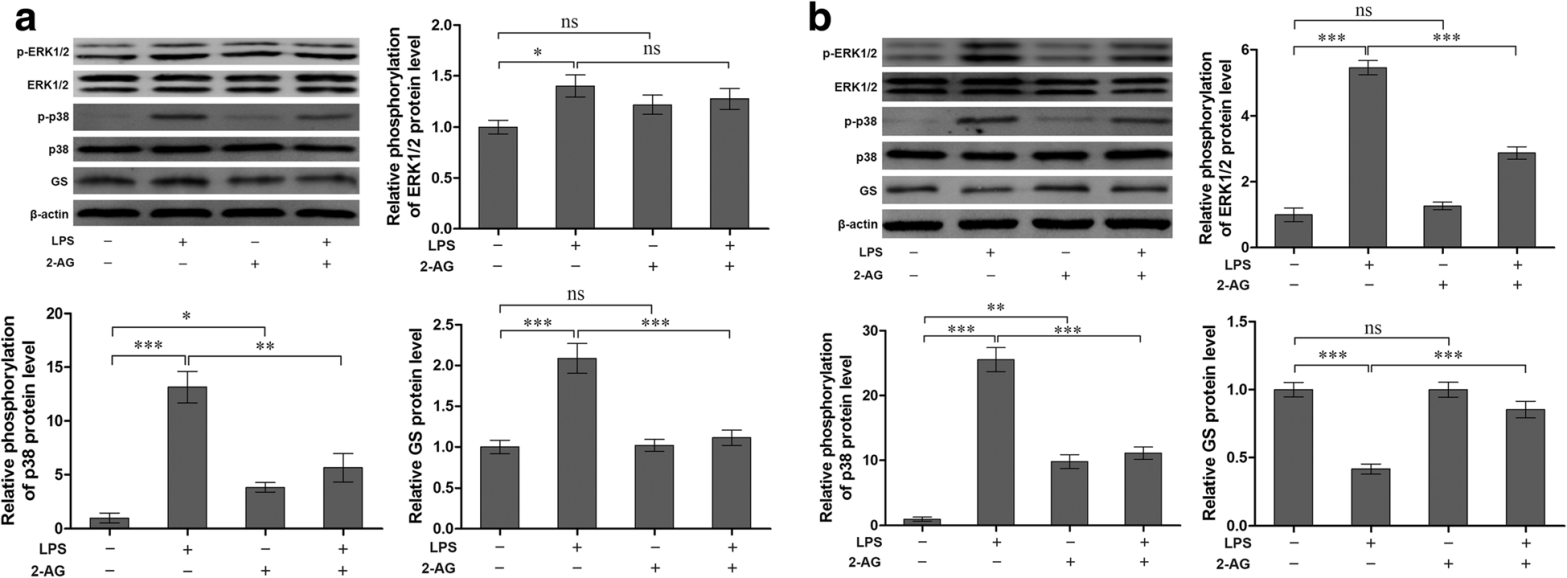
Effects of 2-AG on phosphorylation of p38 (**a**), ERK1/2 (**b**), and GS expression (**a**, **b**) induced by LPS. Astrocytes were pretreated with 0.01 μM 2-AG for 2 h and exposed to 1 μg/ml LPS for 2 h (**a**) or 12 h (**b**). The protein level of GS, p-p38 and p-ERK1/2 were measured, respectively (**a**, **b**). Error bars were ± SD. *n* = 3. *p < 0.05, **p < 0.01, and ***p < 0.001

To investigate the effects of 2-AG on activation of astrocytes induced by the early phase of LPS exposure, the cells were pretreated with 0.01 μM 2-AG for 2 h and/or 1 μg/ml LPS for 2 h. Compared with control, exposure of astrocytes to LPS significantly elevated the expressions of p-ERK1/2 (1.40 ± 0.11 for LPS vs. 1.00 ± 0.07 for control, *p <* 0.05), p-p38 (13.14 ± 1.47 for LPS vs. 1.00 ± 0.45 for control, *p* < 0.001), and GS (2.09 ± 0.18 for LPS vs. 1.00 ± 0.08 for control, *p <* 0.001). 2-AG could significantly reverse the LPS-induced changes of p-p38 (5.64 ± 1.31 for 2-AG plus LPS vs. 13.14 ± 1.47 for LPS, *p <* 0.01) and GS (1.11 ± 0.09 for 2-AG plus LPS vs. 2.09 ± 0.18 for LPS, *p <* 0.001) when compared with LPS alone group, but no significant change was observed for the p-ERK1/2 expression (Fig. 4a). In addition, 2-AG alone could significantly activate p38 (3.84 ± 0.44, *p <* 0.05), but not ERK1/2 (Fig. 4a).

### 2-AG suppressed activation of ERK1/2 and p38, and downregulation of GS induced by the late phase of LPS exposure

Similarly, the effects of 2-AG on the activation of astrocytes induced by the late phase of LPS exposure were investigated. Astrocytes were pretreated with 0.01 μM 2-AG for 2 h and/or 1 μg/ml LPS for 12 h (Fig. 4b). Treatment of astrocytes with LPS for 12 h significantly increased the phosphorylation levels of ERK1/2 (5.45 ± 0.22 for LPS vs. 1.00 ± 0.21 for control, *p <* 0.001) and p38 (25.53 ± 1.85 for LPS vs. 1.00 ± 0.31 for control, *p <* 0.001). Pretreatment with 2-AG significantly attenuated LPS-induced phosphorylation of ERK1/2 (2.87 ± 0.19 for 2-AG plus LPS vs. 5.45 ± 0.22 for LPS, *p <* 0.001) and p38 (11.10 ± 0.95 for 2-AG plus LPS vs. 25.53 ± 1.85 for LPS, *p <* 0.001). Stimulation of astrocytes by LPS for 12 h significantly decreased the expression level of GS (0.42 ± 0.04 for LPS vs. 1.00 ± 0.05 for control, *p <* 0.001) and pretreatment with 2-AG reversed LPS-induced downregulation of GS expression (0.85 ± 0.06 for 2-AG plus LPS vs. 0.42 ± 0.04 for LPS, *p <* 0.001).

### 2-AG inhibited the translocations of MAPK and GS changes induced by LPS exposure

**Fig. 5:**
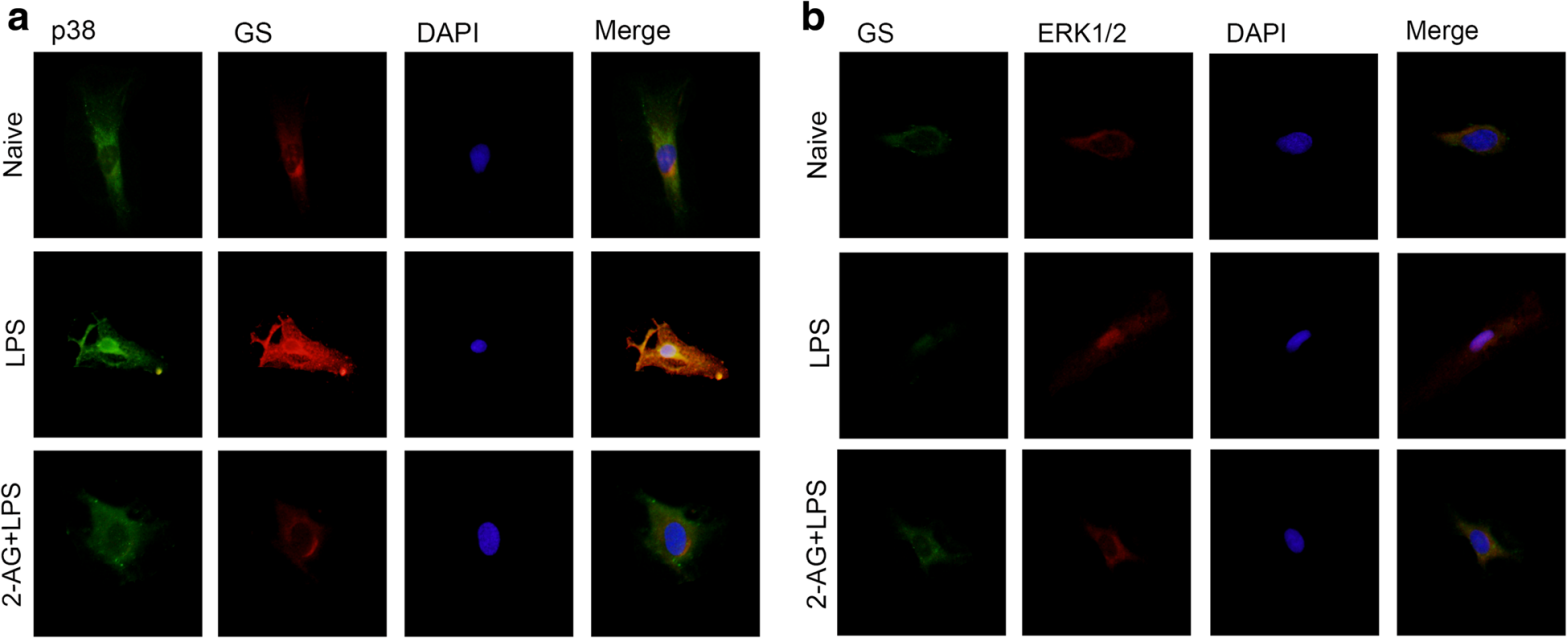
Effects of 2-AG on translocation of p38 (**a**), ERK1/2 (**b**), and expression of GS (**a**, **b**) in astrocytes induced by LPS. Astrocytes were pretreated with 0.01 μM 2-AG for 2 h and exposed to 1 μg/ml LPS for 2 h (**a**) or 12 h (**b**). Immunocytochemistry assay was used to analyze the translocation of p38 and ERK1/2 and the change of GS expression. Scar bar = 10 μm

Previous studies have demonstrated that p38 performs effects by translocating from cytoplasm to nucleus [17, 33]. To investigate the effects of 2-AG on p38 translocation accompanying GS upregulation induced by the early phase of LPS exposure, astrocytes were pretreated with 0.01 μM 2-AG for 2 h and then exposed to 1 μg/ml LPS for 2 h, and p38 translocation and GS expression change were assessed using immunostaining assay (Fig. 5a). The results showed that pretreatment with 2-AG inhibited the nuclear translocation of p38 and cytoplasmic GS upregulation induced by early LPS exposure.

Similarly, to investigate the effects of 2-AG on ERK1/2 translocation accompanying GS downregulation induced by the late phase of LPS exposure, astrocytes were pretreated with 0.01 μM 2-AG for 2 h and then exposed to 1 μg/ml LPS for 12 h, and the changes of ERK1/2 translocation and GS expression were assessed using immunostaining assay (Fig. 5b). The results showed that pretreatment with 2-AG inhibited the nuclear translocation of ERK1/2 and cytoplasmic GS downregulation induced by late LPS exposure.

### 2-AG inhibited upregulation of GS expression induced by the early phase of LPS exposure via CB_2_R-p38 pathway

**Fig. 6:**
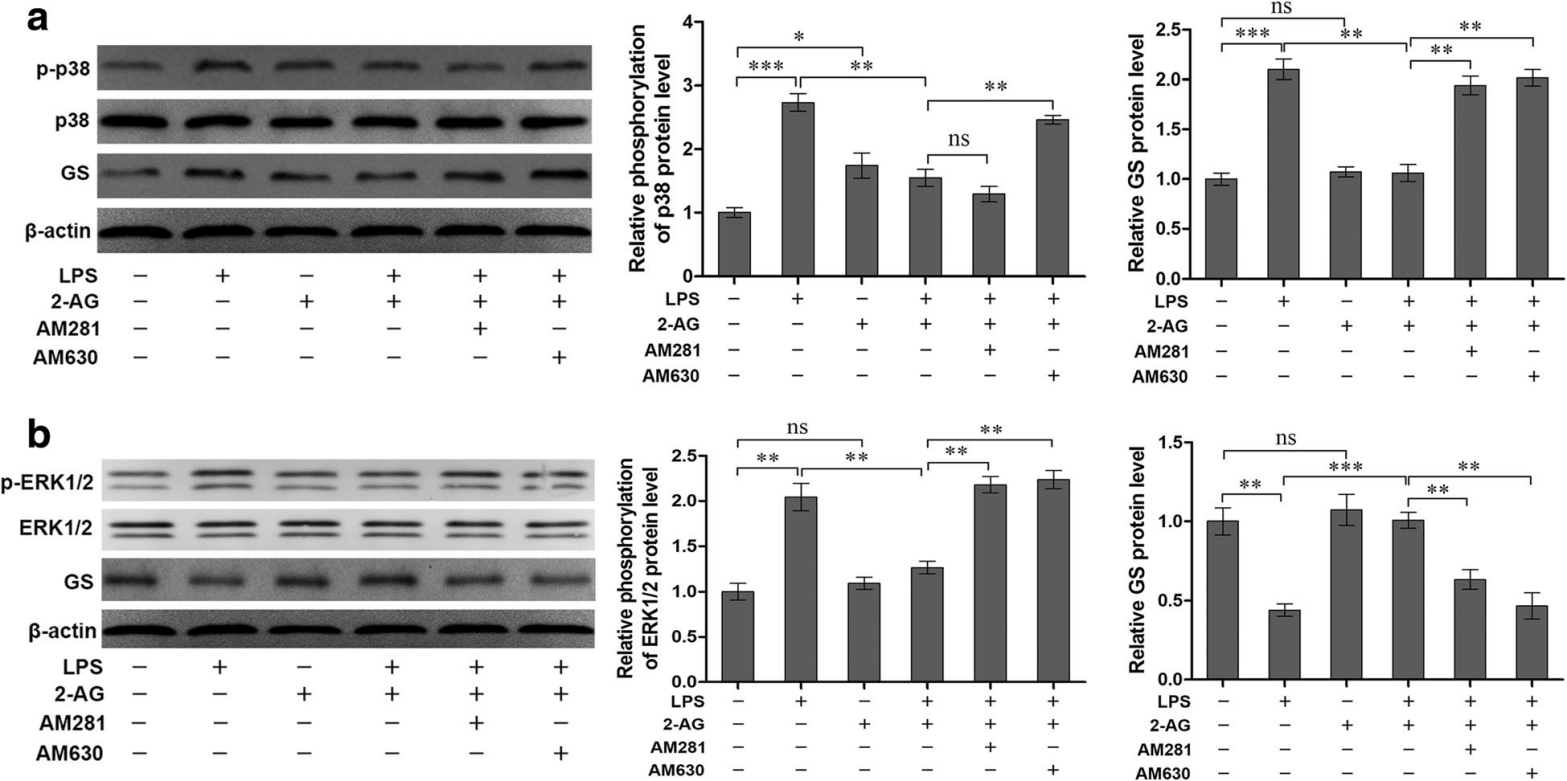
Effects of inhibitors of CB_1_R and CB_2_R antagonists on phosphorylation of p38 (**a**), ERK1/2 (**b**), and GS expression (**a**, **b**) in astrocytes induced by LPS. Astrocytes were pretreated with 1 μM AM281 or AM630 for 1 h before cells were treated with 2-AG and LPS. The protein level of p-p38 (**a**), p-ERK1/2 (**b**), and GS were measured by western blotting. Error bars were ± SD. *n* = 3. *p < 0.05, **p < 0.01, and ***p < 0.001

According to the above results, 2-AG could suppress the upregulation of GS induced by the early phase of LPS exposure via inhibiting the phosphorylation and translocation of p38. It has been demonstrated that two cannabinoid receptors, CB_1_R and CB_2_R, were involved in the pharmacological effects of 2-AG on glial cells [23]. To investigate which receptor mediates the modulation of GS by 2-AG, AM281 and AM630 were used to block the CB_1_R and CB_2_R, respectively. The data showed that treatment of astrocytes with 1 μM AM630 for 1 h significantly reversed the effects of 2-AG on the phosphorylation of p38 (2.46 ± 0.07 for AM630 plus 2-AG and LPS vs. 1.55 ± 0.13 for 2-AG plus LPS, *p <* 0.01) and increase of GS (2.02 ± 0.08 for AM630 plus 2-AG and LPS vs. 1.06 ± 0.09 for 2-AG plus LPS, *p <* 0.01) induced by the early phase of LPS exposure (Fig. 6a). CB_1_R antagonist, AM281, also could reverse the effect of 2-AG on GS upregulation (1.94 ± 0.09 for AM281 plus 2-AG and LPS vs. 1.06 ± 0.09 for 2-AG plus LPS, *p <* 0.01), but not on phosphorylation of p38 induced by early LPS exposure (1.29 ± 0.12 for AM630 plus 2-AG and LPS vs. 1.55 ± 0.13 for 2-AG plus LPS, *p* > 0.05) (Fig. 6a). These data suggested that CB_2_R plays a significant role in the effect of 2-AG via p38-dependent signaling pathway, and activation of CB_1_R also could inhibit the increased GS expression via other uncertain signaling cascades.

### 2-AG reversed downregulation of GS induced by late LPS exposure via CB_1_R/CB_2_R-ERK1/2 pathways

As shown in Fig. 6b, the effects of the late phase of LPS exposure on phosphorylation of ERK1/2 was reversed by 2-AG (1.27 ± 0.07 for 2-AG plus LPS vs. 2.14 ± 0.15 for LPS, *p <* 0.01) while the effects of 2-AG was blocked by CB_1_R antagonist AM281 (2.18 ± 0.09 for AM281 plus 2-AG and LPS vs. 1.27 ± 0.07 for 2-AG plus LPS, *p <* 0.01). Similarly, the effect of the late phase of LPS exposure on GS downregulation was reversed by 2-AG (1.01 ± 0.05 for 2-AG plus LPS vs. 0.44 ± 0.04 for LPS, *p <* 0.001) and the effect of 2-AG was blocked by CB_1_R antagonist AM281 (0.63 ± 0.06 for AM281 plus 2-AG and LPS vs. 1.01 ± 0.05 for 2-AG plus LPS, *p <* 0.01). In addition, CB_2_R antagonist AM630 attenuated the effect of 2-AG on ERK1/2 phosphorylation (2.24 ± 0.10 for AM630 plus 2-AG and LPS vs. 1.27 ± 0.07 for 2-AG plus LPS, *p <* 0.01) and GS downregulation (0.47 ± 0.08 for AM630 plus 2-AG and LPS vs. 1.01 ± 0.05 for 2-AG plus LPS, *p <* 0.01; Fig. 6b) induced by the late phase of LPS exposure.

## Discussion

GS plays a key role through preventing the excessive accumulation of ammonia and glutamate in synaptic surroundings, and thereby suppressing the development of glutamate/ammonia neurotoxicity [2, 34, 35]. Accumulating evidence demonstrated that alterations of GS expression in astrocytes are involved in a number of neurological disorders, including neuropathic pain [3, 5, 7], inflammatory pain [36], Alzheimer’s disease, and Parkinson’s disease [6, 8]. Intriguingly, some diseases, such as hepatic encephalopathy, traumatic brain injury, and epilepsy, demonstrated both increase and decrease of GS while controlling GS expression can alleviate these diseases [4]. Consistently, the present study confirmed a biphasic change of GS expression in astrocytes after treatment with LPS. In addition, the present study found that LPS exposure induced increased of apoptosis and decrease of cell viability in the late stage of LPS exposure. These studies suggest that LPS induces a dynamic change of GS expression in astrocytes following the prolongation of LPS exposure time, which could modulate the survival of astrocytes. An improved understanding of the exact role of GS and mechanism in these neurological disorders and its involvement in pathology of diseases could enable the identification of innovative drugs to treat these diseases.

MAPK signaling, including p38 and ERK1/2, is a family of kinases involved in multiple physiological and pathological processes, including pain and neurodegenerative diseases [17–19]. In addition to neurons, MAPK signaling also exists in astrocytes and is activated under pathological stimulation [20–22]. The present study indicated that p38 and ERK1/2 were activated and translocated into nucleus in astrocytes by LPS exposure with different patterns, i.e., p38 was firstly activated at 30 min and reached peak at 2 h while ERK1/2 activated at 1 h and reached peak at 3 h, while inhibition of p38 and ERK1/2 attenuated the changes of GS expression induced by LPS. These results suggested that there is a sequential activation of MPAK signaling in astrocytes, which plays an important role in modulating the GS expression. In addition, the present results were also consistent with previous study indicating that MAPK signaling is involved in the LPS-induced changes of GS in astrocytes [21].

2-AG is an endocannabinoid playing its roles in binding to CB_1_R and CB_2_R which are found to be expressed in astrocytes [23]. The present study indicated that 2-AG could modulate the GS expression and MAPK activation induced by LPS exposure through different CB receptors. Pascal et al. observed that 2-AG could activate p38 while blockade of CB_1_R could inhibit the effect of 2-AG on p38 [27], suggesting that 2-AG could modulate MAPK signaling in astrocytes. The results of present study indicated that, under the condition of the early phase of LPS exposure, activated p38 could translocate into nucleus, resulting in the increase of GS expression, and 2-AG could suppress the increase of GS by inhibiting the phosphorylation level and translocation of p38. While under the condition of late LPS exposure, activated ERK1/2 translocated into nucleus resulting in decrease of GS expression and 2-AG reversed the decrease of GS expression through reducing the activation and translocation of ERK1/2. It should be noted that although ERK1/2 and p38 were activated by early LPS exposure and late LPS exposure, respectively, the activation was relatively weaker when compared to late and early LPS exposure, respectively. These results indicated that 2-AG modulates the astrocyte survival by modulating p38 and ERK1/2 activation through different CB receptors, which is supported by the results that 2-AG could prevent the apoptosis of astrocytes induced by LPS exposure.

## Conclusion

**Fig. 7:**
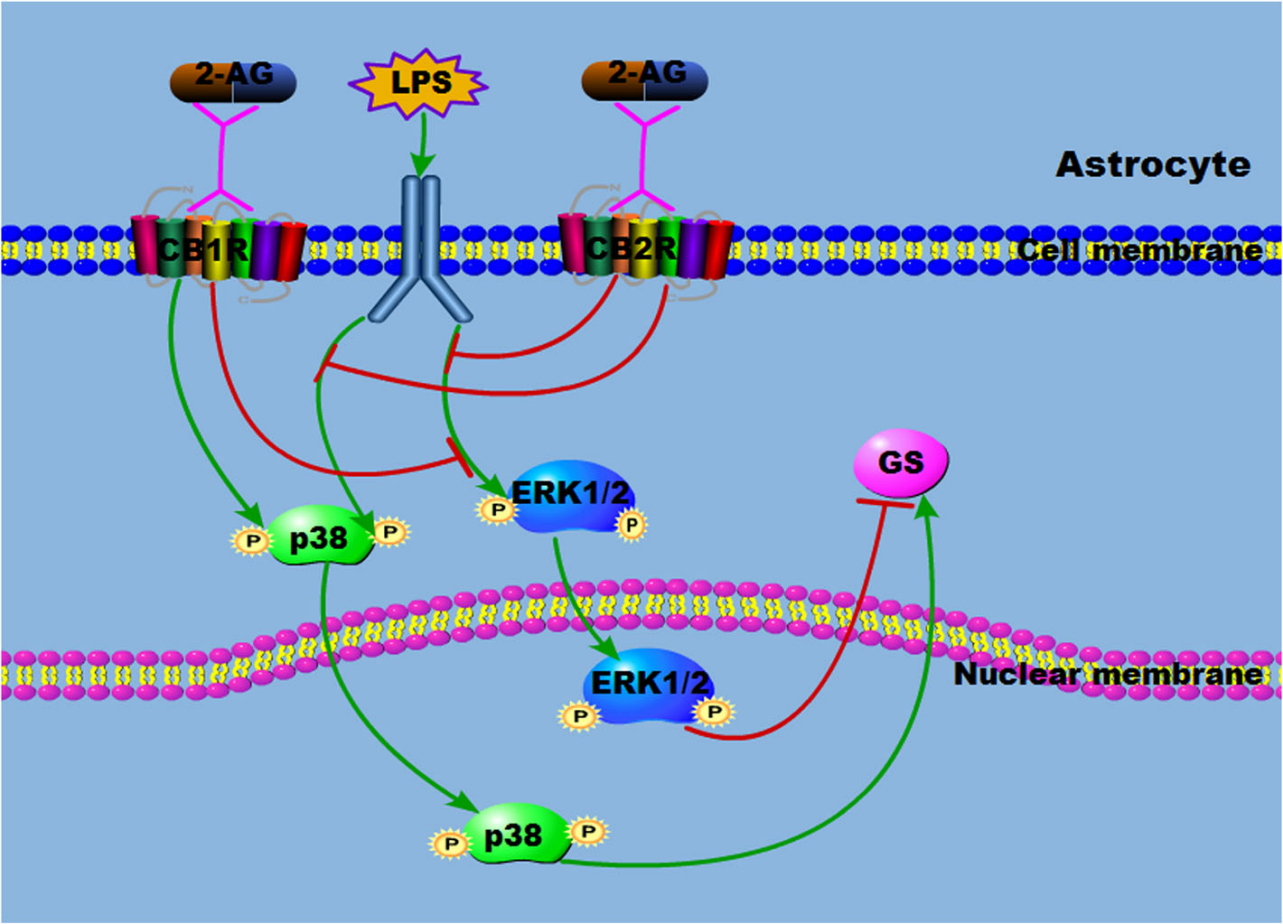
Schematic drawing of the 2-AG on the GS expression and MAPK activation in astrocytes induced by LPS

The results of the present study indicated that LPS exposure for the short term and long term induced different changes of GS in astrocytes with activation of MAPK signaling, including p38 and ERK1/2 (Fig. 7). Endocannabinoid 2-AG modulates the GS expression induced by LPS exposure through p38 and ERK1/2 activation via different CB receptors to display the neuroprotection.

## Abbreviations

2-AG: 2-arachidonyl glycerol
CBR: Cannabinoid receptor
ERK1/2: Extracellular signal-regulated protein kinase 1/2
GFAP: Glial fibrillary acidic protein
GS: Glutamine synthetase
LPS: Lipopolysaccharide
MAPK: Mitogen-activated protein kinase
MTT: Methyl thiazolyl tetrazolium
